# The Effect of the COVID-19 Pandemic on Total Hip and Knee Arthroplasty Surgical Volume in 2020 in Poland

**DOI:** 10.3390/ijerph18168830

**Published:** 2021-08-21

**Authors:** Maria Czubak-Wrzosek, Jarosław Czubak, Dariusz Grzelecki, Marcin Tyrakowski

**Affiliations:** 1 Department of Spine Disorders and Orthopedics, Centre of Postgraduate Medical Education, Gruca Orthopaedic and Trauma Teaching Hospital, Konarskiego 13, 05-400 Otwock, Poland; marcintyrak@gmail.com; 2 Department of Orthopedics, Pediatric Orthopedics and Tramatology, Centre of Postgraduate Medical Education, Gruca Orthopaedic and Trauma Teaching Hospital, Konarskiego 13, 05-400 Otwock, Poland; czubakjarek@gmail.com; 3 Department of Orthopedics and Rheumoorthopedics, Centre of Postgraduate Medical Education, Gruca Orthopaedic and Trauma Teaching Hospital, Konarskiego 13, 05-400 Otwock, Poland; dariusz.grzelecki@gmail.com

**Keywords:** arthroplasty, COVID-19, total hip arthroplasty, total knee arthroplasty, revision arthroplasty

## Abstract

The aim of this study was to analyse the effect of the first year of the COVID-19 pandemic on total hip arthroplasty (THA) and total knee arthroplasty (TKA) surgical volume in Poland. A retrospective analysis of data concerning THA and TKA collected by the National Health Fund in Poland in 2019 and in 2020 has been conducted. The number of primary hip or knee arthroplasties in 2020 was around 71% and 67% of the number registered in 2019, respectively. There was also a decline in the volume of revision arthroplasties observed, with 65% and 63% of THA and TKA revisions performed in 2019. The most significant decrease was observed in April and May, and during the second wave of the pandemic in November 2020, with a decline of 87%, 55% and 56%, respectively. The results of this study show the significant impacts that the COVID-19 pandemic had on the volume of elective hip and knee arthroplasties in Poland in 2020. In comparison with 2019, a decrease of around 30% for primary and of 40% for revision arthroplasties was observed. The most significant decline was observed in April and May 2020, and during the second wave of the COVID-19 pandemic in Poland in November 2020.

## 1. Introduction

Total hip arthroplasty (THA) and total knee arthroplasty (TKA) have been proven as the [1] methods of choice in the treatment of end-stage hip or knee pathologies [2,3,4,5]. Improvement in biomaterials, surgical techniques and implant designs has been the reason for more indications for THA and TKA [1,3,4,6,7,8,9].

Thus, the number of total joint arthroplasties has been increasing in the recent decades [10,11,12].

A steady increase in volume of THA and TKA in the United States has been projected up to 2030, with no negative influence of economic downturns in the 2000s [11,12,13,14,15].

The vast majority of THA and TKA procedures in Poland are covered by the National Health Fund (NFZ—Narodowy Fundusz Zdrowia), even in private hospitals. The number of both surgical procedures in Poland has been increasing, similarly to data reported for other countries [16,17].

COVID-19 infection was announced as a global pandemic by the World Health Organisation on 11 March 2020 [18]. The first confirmed case of SARS-Cov-2 infection in Poland was reported on 4 March 2020, and on 13 March, the Polish government implemented regulations reducing the functioning of most institutions and enterprises. There were 21 hospitals around Poland transformed into centres dedicated to the treatment of patients with COVID-19 infections. These hospitals were providing comprehensive care to patients infected with COVID-19, including the orthopaedic and traumatology departments. In order to prevent the health system overload and to provide the best care for SARS-CoV-2-infected patients, other medical institutions were recommended to reduce the number of elective admissions and surgical procedures. The cancellation of non-urgent elective surgeries severely affected the number of orthopaedic procedures, including THA and TKA. Due to a relatively low number of COVID-19 cases in Poland, elective procedures were progressively resumed in June 2020. In fall, the number of COVID-19 cases increased, however no official recommendations regarding elective orthopaedic surgeries were released.

To date, the real impact of the COVID-19 pandemic on THA and TKA in Poland has not been published.

The aim of this study was to analyse the effect of the first year of the COVID-19 pandemic on THA and TKA surgical volume in Poland.

## 2. Materials and Methods

This study was approved by the Institutional Review Board (protocol #nr 45/2021). A retrospective analysis of the registry data of THA and TKA funded by the National Health Fund in Poland in 2019 and in 2020 has been conducted. The data were collected by and obtained from NFZ.

The total numbers of the hip and knee arthroplasties performed in 2019 and 2020 have been compared. The volume of THA and TKA performed in the particular months was analysed and compared between 2019 and 2020. The analyses were taken for the total number of THA and TKA, THA or TKA separately and for the primary and revision THA and TKA, respectively.

Descriptive statistics and graphs (Microsoft Excell 365; Redmont, WA, USA) were used to present the data.

## 3. Results

The total number of both THA and TKA was 93,022 and 64,791 in 2019 and 2020, respectively (Table 1). The number of surgical procedures in 2020 ranged from 63% to 71% compared to 2019 (Table 1).

**Figure 1:**
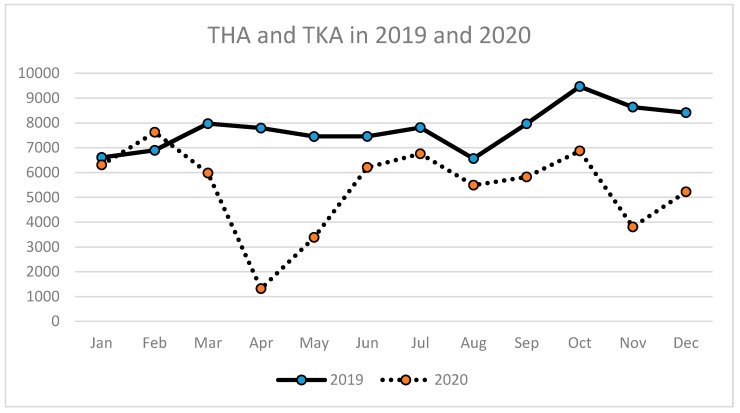
Number of total hip arthroplasties (THA) and total knee arthroplasties (TKA) performed in the particular months of 2019 and 2020 in Poland.

The number of TKA and THA performed in the particular months of 2019 and 2020 is presented in Table 2. The decrease in arthroplasty surgical volume in 2020 compared to 2019 has been observed since March, with the lowest number of surgeries in April and May, with a subsequent gradual increase within the next 4 months and a drop in November with the second wave of the COVID-19 pandemic (Table 2, Figure 1).

**Figure 2:**
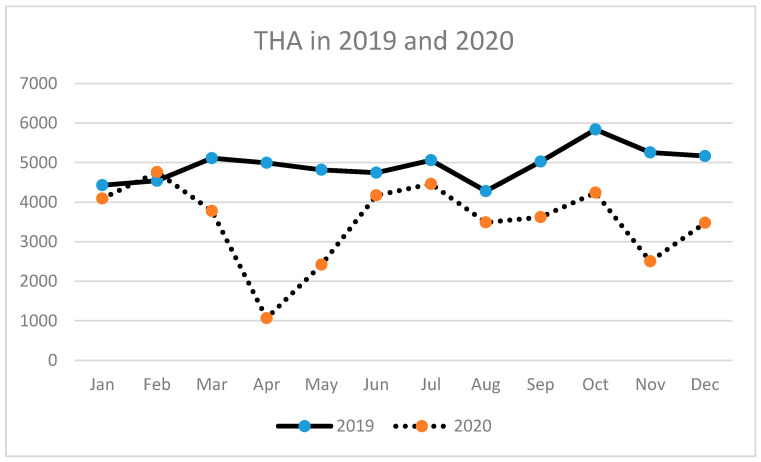
Number of total hip arthroplasties (THA) performed in the particular months of 2019 and 2020 in Poland.

**Figure 3:**
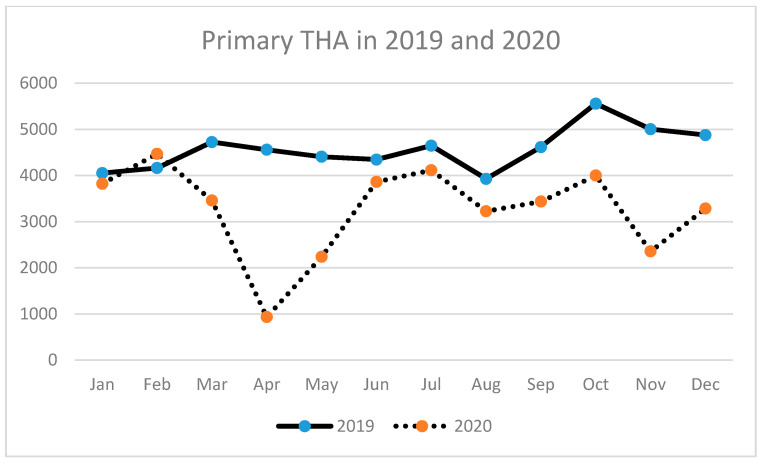
Number of primary total hip arthroplasties (THA) performed in the particular months of 2019 and 2020 in Poland.

**Figure 4:**
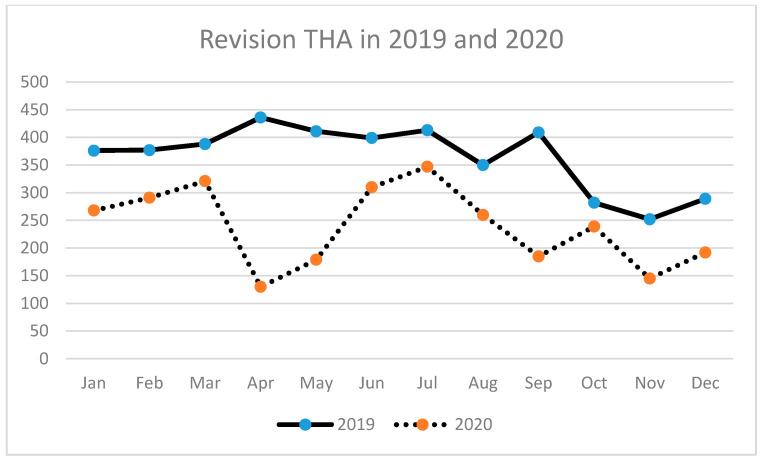
Number of revision total hip arthroplasties (THA) performed in the particular months of 2019 and 2020 in Poland.

**Figure 5:**
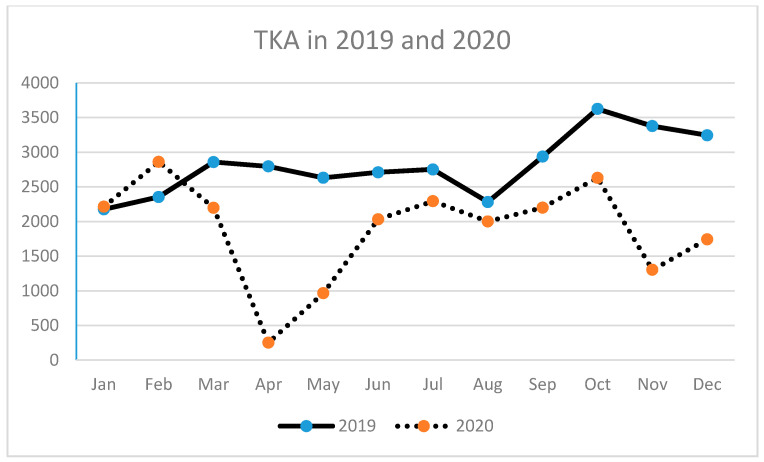
Number of total knee arthroplasties (TKA) performed in the particular months of 2019 and 2020 in Poland.

**Figure 6:**
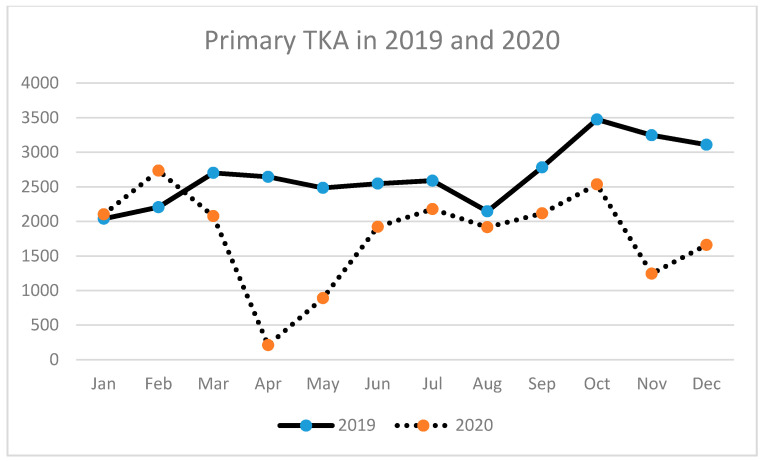
Number of primary total knee arthroplasties (TKA) performed in the particular months of 2019 and 2020 in Poland.

**Figure 7:**
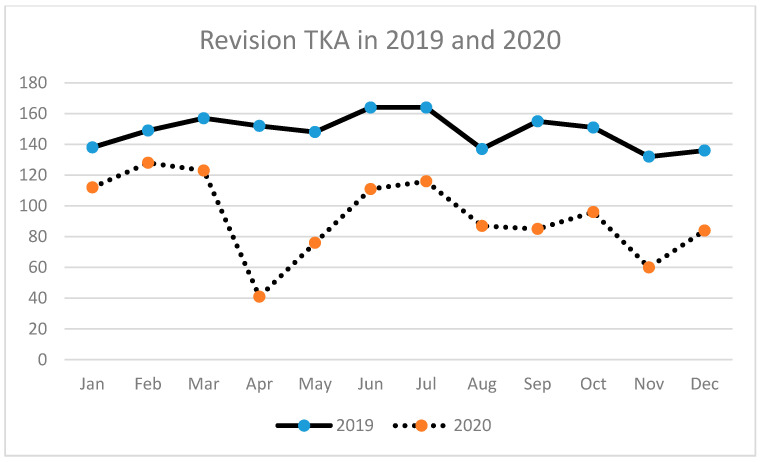
Number of revision total knee arthroplasties (TKA) performed in the particular months of 2019 and 2020 in Poland.

A similar tendency has been observed for THA (primary and revision) (Table 3, Figure 2, Figure 3 and Figure 4) as well as for TKA (primary and revision) (Table 4, Figure 5, Figure 6 and Figure 7).

## 4. Discussion

In this study, we present an analysis of the real impact of the COVID-19 pandemic on the number of THA and TKA in Poland. Such an analysis has not yet been published. COVID-19 had a significant effect on the hip and knee arthroplasty surgical volume in the first year of the pandemic in Poland. According to our analysis of data collected by the NFZ, the numbers of primary THA and TKA in 2020 were around 71% and 67% of the numbers registered in 2019, respectively. The decline in the volume of revision arthroplasties was even greater, with 65% and 63% of THA and TKA revisions performed in 2019.

The most significant decrease in the number of procedures was observed in April and May 2020, which was at the time of the strictest lockdown regulations in Poland. The relatively low number of cases during the first wave of the pandemic enabled Polish hospitals to resume elective surgical procedures, which resulted in higher numbers of arthroplasty procedures between June and September 2020. The second wave of the pandemic in the fall of 2020 resulted again in a decrease in the number of procedures, with the second lowest amount observed in November 2020, in accordance with the peak number of COVID-19 cases in Poland.

The impact of COVID-19 on arthroplasty was observed in many countries all around the world. According to Yapp et al., who analysed the data from the Scottish Arthroplasty Project, the number of primary THA and TKA procedures during the COVID-19 pandemic in Scotland fell by 53.6% and 61.1%, respectively. After resuming elective surgical procedures, Scottish hospitals achieved only 40–50% of the previous monthly volume [19]. An even bigger decrease of arthroplasty volume was observed at the beginning of the epidemic in the United States of America. Barnes et al. reported a significant decline in TKA and THA surgical volumes in mid-March 2020, of 94% and 92%, respectively [20]. According to Chia-Lung Shih et al., even though the epidemiological situation in Taiwan differed from that observed in the US or Scotland, with a very small number of COVID-19 cases and no restrictions concerning the elective surgical procedures, the volume of orthopaedic surgeries decreased by 20–30%. The decrease in orthopaedic surgical procedures in Taiwan in March and April 2020 could be associated with the patients’ fear of COVID-19 infection [21]. According to a survey conducted among the members of the European Hip Society and the European Knee Associates in April 2020, the procedures mostly disrupted by COVID-19 were primary total joint arthroplasty (92.6% cancelled) and aseptic revisions (94.7% cancelled), while 87.2% of periprosthetic fractures, 75.8% of hip arthroplasty for femoral neck fractures and 75.8% of septic revisions for acute infections were performed [22].

In the study by Clement et al., perioperative COVID-19 infection doubled the risk of postoperative mortality in orthopaedic and trauma patients in the UK, although most COVID-19 infections were observed in trauma patients (60 patients), with only 2 in elective orthopaedic procedures [23].

The results of our study confirm how large of an impact COVID-19 had on the joint arthroplasty volume in 2020. The large number of cancelled or postponed surgeries resulted in significant financial losses of medical institutions, changes in clinical practice of orthopaedic surgeons, as well as a profound effect on the physical, psychological and financial status of the patients awaiting the joint replacement [24,25,26,27,28,29]. Furthermore, younger patients with higher pain levels and worse joint function were more eager to proceed with the surgery, despite the pandemic [27,29]. Despite the impaired quality of life, most patients awaiting total joint arthroplasty (TJA) understood the need for the surgery delay due to the pandemic and said it was in their best interest [30].

The COVID-19 pandemic not only caused diminution of surgical procedures, but also severely impaired the post-operative care, including physical rehabilitation and surgeons’ follow-ups [25].

Several articles have discussed the resuming of elective joint arthroplasties during the COVID-19 pandemic [31,32,33,34]. Guidelines included reorganisation of hospital wards, patients’ selection and preoperative, perioperative and postoperative management. In the initial phase after resuming elective procedures, it was recommended to operate on patients with good general health status (ASA I and II) using spinal or regional anaesthesia, minimise the surgical and hospitalisation time, use absorbable sutures for wound closure, include telemedicine in the patient’s follow-up and provide a home care physical therapy [31,34]. Nevertheless, restarting and achieving the pre-pandemic volume of elective TJA has been a challenge for orthopaedic surgeons all around the world. According to our data, the gap between the number of arthroplasties performed in Poland in 2020 and 2019 was 28,231. Concerning the increasing trend in arthroplasty procedures in Poland, the real number of patients awaiting joint replacement was probably higher. Assuming 110% of pre-pandemic productivity, it will take around 3 years to catch up with the demand. According to other studies, it is projected that even with 120% pre-COVID productivity, the catch up will take from 1 to 4 years [19,35].

A potential limitation of our study is in the paucity of data available in the registry we used for the analyses. It might be valuable to perform detailed analyses including the age or sex of patients operated on in 2019 and in 2020. Furthermore, the correlations with the type of the hospital or specific region of Poland and the number of TKA and THA in the specific years would be of great value. However, to date, these data are still not available.

## 5. Conclusions

In summary, our results show the significant impact that the COVID-19 pandemic had on the volume of elective hip and knee arthroplasty in Poland. In comparison with 2019, a decrease of around 30% for primary arthroplasties and an even larger decrease of 40% for revision THA and TKA were observed in the first year of the pandemic. The most significant decrease in the volume of arthroplasties was observed in April and May 2020, and during the second wave of the COVID-19 pandemic in Poland in November 2020. Restoring previous capacity and catching up poses a great challenge for the healthcare system and all orthopaedic surgeons.

## Figures and Tables

**Table 1:** Numbers of total hip arthroplasties (THA) and total knee arthroplasties (TKA) performed in 2019 and 2020 in Poland.

Procedure	Year		
	2019	2020	2020/2019 (%)
THA + TKA	93,022	64,791	70
THA	59,277	42,089	71
TKA	33,745	22,702	67
Primary THA	54,895	39,222	71
Primary TKA	31,962	21,583	68
Revision THA	4382	2867	65
Revision TKA	1783	1119	63

**Table 2:** Number of total hip arthroplasties (THA) and total knee arthroplasties (TKA) performed in the particular months of 2019 and 2020 in Poland.

	Number of THA and TKA		
Year	2019	2020	2020/2019 (%)
Month			
**January**	6606	6306	95
**Februrary**	6895	7624	111
**March**	7972	5979	75
**April**	7791	1318	17
**May**	7451	3385	45
**June**	7456	6207	83
**July**	7813	6757	86
**August**	6561	5490	84
**September**	7964	5823	73
**October**	9465	6871	73
**November**	8636	3808	44
**December**	8412	5223	62

**Table 3:** Number of total hip arthroplasties (THA) performed in the particular months of 2019 and 2020 in Poland.

	Total THA			Primary THA			Revision THA		
Year	2019	2020	2020/2019 (%)	2019	2020	2020/2019 (%)	2019	2020	2020/2019 (%)
Month									
**January**	4430	4092	92	4054	3824	94	376	268	71
**Februrary**	4541	4762	105	4164	4471	107	377	291	77
**March**	5114	3780	74	4726	3459	73	388	321	83
**April**	4995	1066	21	4559	936	21	436	130	30
**May**	4819	2419	50	4408	2240	51	411	179	44
**June**	4746	4174	88	4347	3864	89	399	310	78
**July**	5061	4463	88	4648	4116	89	413	347	84
**August**	4279	3488	82	3929	3228	82	350	260	74
**September**	5027	3622	72	4618	3437	74	409	185	45
**October**	5840	4240	73	5558	4001	72	282	239	85
**November**	5258	2504	48	5006	2359	47	252	145	58
**December**	5167	3479	67	4878	3287	67	289	192	66

**Table 4:** Number of total hip arthroplasties (THA) performed in the particular months of 2019 and 2020 in Poland.

	Total THA			Primary THA			Revision THA		
Year	2019	2020	2020/2019 (%)	2019	2020	2020/2019 (%)	2019	2020	2020/2019 (%)
Month									
**January**	2176	2214	102	2038	2102	103	138	112	81
**Februrary**	2354	2862	122	2205	2734	124	149	128	86
**March**	2858	2199	77	2701	2076	77	157	123	78
**April**	2796	252	9	2644	211	8	152	41	27
**May**	2632	966	37	2484	890	36	148	76	51
**June**	2710	2033	75	2546	1922	75	164	111	68
**July**	2752	2294	83	2588	2178	84	164	116	71
**August**	2282	2002	88	2145	1915	89	137	87	64
**September**	2937	2201	75	2782	2116	76	155	85	55
**October**	3625	2631	73	3474	2535	73	151	96	64
**November**	3378	1304	39	3246	1244	38	132	60	45
**December**	3245	1744	54	3109	1660	53	136	84	62

## Data Availability

The data presented in this study are available upon request from the corresponding author and online: Publikacje/O NFZ/Narodowy Fundusz Zdrowia (NFZ)—finansujemy zdrowie Polaków.
